# Bis(μ-phenyl­tellurido-κ^2^ *Te*:*Te*)bis­[tetra­carbonyl­rhenium(I)]

**DOI:** 10.1107/S1600536810014297

**Published:** 2010-04-24

**Authors:** J. Muthukumaran, M. Kannan, A. Vanitha, Bala Manimaran, R. Krishna

**Affiliations:** a Centre for Bioinformatics, Pondicherry University, Puducherry 605 014, India; b Department of Chemistry, Pondicherry University, Puducherry 605 014, India

## Abstract

The title compound, [Re_2_(C_6_H_5_Te)_2_(CO)_8_], crystallizes with two mol­ecules in the asymmetric unit, in which two Re atoms are coordinated in a slightly distorted octa­hedral environment and are bridged by two Te atoms, which show a distorted trigonal-pyramidal geometry. The torsion angles for the Te—Re—Te—Re sequence of atoms are 19.29 (18) and 16.54 (16)° in the two mol­ecules. Thus, the Re—Te four-membered rings in the two mol­ecules deviate significantly from planarity. Two intra­molecular C—H⋯O inter­actions occur in one of the mol­ecules. Te—Te [4.0551 (10) Å] inter­actions between the two mol­ecules and weak inter­molecular C—H⋯O inter­actions stabilize the crystal packing.

## Related literature

For the biological importances of Re and Te compounds, see: Begum *et al.* (2008 ▶); Atwood *et al.* (1983 ▶); Zhang & Leong (2000 ▶); Lima *et al.* (2009 ▶); Cunha *et al.* (2009 ▶); Kopf-Maier & Klapötke (1992 ▶); Cerecetto *et al.* (1997 ▶). For a related structure, see: Cecconi *et al.* (1998 ▶). For a structure with weak Te⋯Te contacts, see: Ritch & Chivers (2009 ▶). For puckering analysis, see: Cremer & Pople (1975 ▶)
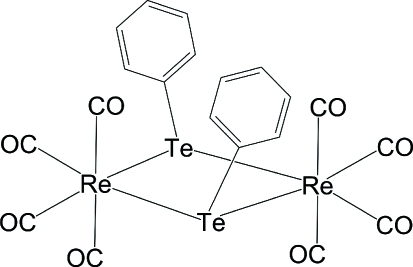


## Experimental

### 

#### Crystal data

[Re_2_(C_6_H_5_Te)_2_(CO)_8_]
*M* *_r_* = 2011.76
Triclinic, 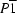

*a* = 9.8062 (13) Å
*b* = 16.3418 (15) Å
*c* = 17.1000 (14) Å
α = 106.593 (7)°
β = 99.932 (9)°
γ = 105.572 (10)°
*V* = 2435.9 (4) Å^3^
*Z* = 2
Mo *K*α radiation
μ = 12.32 mm^−1^
*T* = 150 K
0.32 × 0.28 × 0.22 mm

#### Data collection

Oxford Diffraction Xcalibur-S diffractometer
Absorption correction: multi-scan (*CrysAlis PRO*; Oxford Diffraction, 2009 ▶) *T*_min_ = 0.110, *T*_max_ = 0.173
20451 measured reflections
8557 independent reflections
7388 reflections with *I* > 2σ(*I*)
*R*_int_ = 0.038

#### Refinement

*R*[*F* ^2^ > 2σ(*F* ^2^)] = 0.034
*wR*(*F* ^2^) = 0.079
*S* = 1.06
8557 reflections
577 parameters
H-atom parameters constrained
Δρ_max_ = 2.27 e Å^−3^
Δρ_min_ = −2.55 e Å^−3^

### 

Data collection: *CrysAlis PRO* (Oxford Diffraction, 2009 ▶); cell refinement: *CrysAlis PRO*; data reduction: *CrysAlis PRO*; program(s) used to solve structure: *SHELXS97* (Sheldrick, 2008 ▶); program(s) used to refine structure: *SHELXL97* (Sheldrick, 2008 ▶); molecular graphics: *ORTEP-3 for Windows* (Farrugia, 1997 ▶); software used to prepare material for publication: *PLATON* (Spek, 2009 ▶).

## Supplementary Material

Crystal structure: contains datablocks I, global. DOI: 10.1107/S1600536810014297/sj2772sup1.cif

Structure factors: contains datablocks I. DOI: 10.1107/S1600536810014297/sj2772Isup2.hkl

Additional supplementary materials:  crystallographic information; 3D view; checkCIF report

## Figures and Tables

**Table 1:** Hydrogen-bond geometry (Å, °)

*D*—H⋯*A*	*D*—H	H⋯*A*	*D*⋯*A*	*D*—H⋯*A*
C16*A*—H16*A*⋯O6*A*	0.95	2.74	3.592 (11)	149
C20*A*—H20*A*⋯O2*A*	0.95	2.87	3.678 (12)	143
C12*B*—H12*B*⋯O5*B*^i^	0.95	2.63	3.433 (11)	143

Symmetry code: (i) 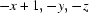.

**Fig. 1.**
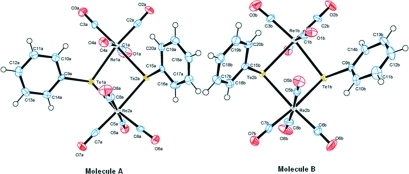
: ORTEP – 3 view of the structure of title compound, showing the atom-labeling scheme. Displacement ellipsoidsare drawn at the 50% probability level.

**Fig. 2.**
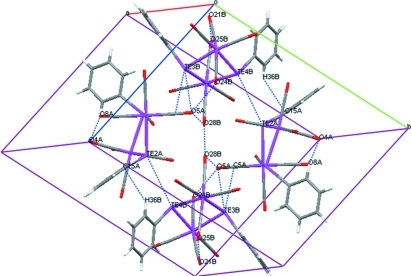
: View of the crystal packing diagram of the title compound in the unit cell.

**Fig. 3.**
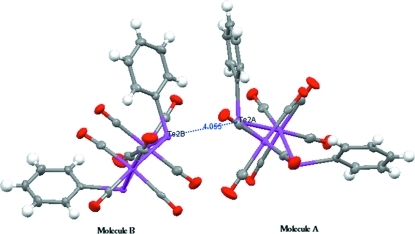
: Crystal structure of title compound showing Te—Te interaction .

## supplementary crystallographic information

### Comment

The biological activities of rhenium and tellurium compounds have been studied and revealed interesting and promising applications (Begum *et al.*, 2008; Atwood *et al.*,  1983; Zhang & Leong, 2000).  Rhenium derivatives have a wide range of biological applications such as antitumor, cytostatic (Kopf-Maier & Klapotke, 1992) and antitrypanosomal activity (Cerecetto *et al.*, 1997). Organo tellurium compounds are the inhibitors of human cathepsin B, which is a highly predictive indicator for prognosis and diagnosis of cancer. Some of the tellurium derivatives exhibit antioxidant and immunomodulatory effects (Cunha *et al.*, 2009). Recently, a novel organotellurium compound-RT01 was proved to act as antileishmanial agent (Lima *et al.*, 2009). In view of these important features we have chosen the title compound for crystal structure analysis.

The title compound was crystallized with two independent molecules (A & B) in the asymmetric unit (Fig. 1), which adopted dinuclear metallacyclic structure, where each rhenium Re(CO)_4_ core is bonded by two phenyl tellurolate groups and hence Re centers attained a distorted octahedral geometry. The r.m.s deviation for four-membered ring with carbonyl atoms of two molecules is 0.016 Å, calculated by Platon - automolfit program (Spek, 2009) and the maximum deviation was observed in phenyl tellurolate groups. The Re—Te bond distances are nearly equal in both the molecules and are similar to those in a related structure (Cecconi *et al.*, 1998). The six atoms (C9A—Te1A—Re2A—Te2A—C15A—Re1A & C15B—Te2B—Re2B—Te1B—C9B—Re1B) generate six-membered rings each with a boat conformation; puckering parameters (Cremer & Pople, 1975) A: q2 = 0.3707(0.0000) Å, q3 = -1.8679 (0.0001) Å, phi2 = 113.59 (0.01)°, QT =1.9043 (0.0001), theta2 = 168.78 (0.00)° and B: q2 = 0.5108(0.0002) Å, q3 = -1.9993(0.0002)Å , phi2 =125.79(0.02)°, QT = 2.0635(0.0003), theta2 = 165.67(0.01)° . The crystal packing (Fig. 2) of the molecule in the unit cell is influenced by C—H···O and Te—Te interactions. The two molecules in the unit cell are interconnected with each other through Te—Te interaction [4.0551 (10) Å] and illustrated in Fig. 3. This Te···Te separation is similar to that observed previously (Ritch & Chivers, 2009). In their study, weak intermolecular Te···Te contacts were observed in the compound C_36_H_84_Cu_3_N_3_O_3_P_6_Te_3_ ranging from 3.891 Å to 4.039 Å. However, the Te···Te separation observed here is smaller than that in NaCuTe, 4.38Å, (Seong *et al.*, 1994). Based on these previous studies, the observed Te···Te contact is significant and contributes to the packing. Intermolecular and interatomic O—O bond distances are also observed for O6B—O4B, O5A—O8B, O5A—O4B, O8B—O8B, O5A—O3A, O1B—O5B, O4A—O8A with distances of 2.928 (9) Å, 2.975 (10) Å, 3.025 (9) Å, 3.014 (13) Å,2.951 (9) Å, 2.9710 (89) Å and 2.9387 (87) Å respectively.

### Experimental

A mixture of Re_2_(CO)_10_ (130 mg, 0.2 mmol) and diphenyl ditelluride (41 mg, 0.1 mmol), 4-phenylpyridine (93 mg, 0.6 mmol) were taken in a 50 ml two neck Schlenk flask and fitted with a reflux condenser. The system was evacuated and purged with N_2_. Freshly distilled mesitylene (30 ml) was added under N_2_ atmosphere. The reaction mixture was heated to 403 K under N_2_ for 6 h and allowed to cool to room temperature. The mesitylene was removed by vacuum distillation and the solid mixture was washed with hexane, chromatographed on silica gel using dichloromethane and hexane (1:9) as eluent to obtain the yellow color solid of without phenylpyridine substituted compound [(CO)_4_Re(µ-TeC_6_H_5_)_2_Re(CO)_4_]. Single crystal of the title compound was obtained by slow diffusion of hexane into a concentrated solution of the title compound in dichloromethane at 278 K.

### Refinement

The hydrogen atoms were placed in calculated positions (C—H = 0.95 Å) and included in the refinement in riding-model approximation with U_ĩso_(H) = 1.2U_eq_(C).

### Crystal data

**Table:**

[Re_2_(C_6_H_5_Te)_2_(CO)_8_]	*Z* = 2
*M**_r_* = 2011.76	*F*(000) = 1792
Triclinic, *P*1	*D*_x_ = 2.743 Mg m^−^^3^
Hall symbol: -P 1	Mo *K*α radiation, λ = 0.71073 Å
*a* = 9.8062 (13) Å	Cell parameters from 15161 reflections
*b* = 16.3418 (15) Å	θ = 2.9–32.6°
*c* = 17.1000 (14) Å	µ = 12.32 mm^−^^1^
α = 106.593 (7)°	*T* = 150 K
β = 99.932 (9)°	Block, yellow
γ = 105.572 (10)°	0.32 × 0.28 × 0.22 mm
*V* = 2435.9 (4) Å^3^	

### Data collection

**Table:**

Oxford Diffraction Xcalibur-S diffractometer	8557 independent reflections
Radiation source: fine-focus sealed tube	7388 reflections with *I* > 2σ(*I*)
graphite	*R*_int_ = 0.038
Detector resolution: 15.9948 pixels mm^-1^	θ_max_ = 25.0°, θ_min_ = 2.9°
ω scans	*h* = −11→11
Absorption correction: multi-scan (*CrysAlis PRO*; Oxford Diffraction, 2009)	*k* = −19→19
*T*_min_ = 0.110, *T*_max_ = 0.173	*l* = −20→20
20451 measured reflections	

### Refinement

**Table:**

Refinement on *F*^2^	Primary atom site location: structure-invariant direct methods
Least-squares matrix: full	Secondary atom site location: difference Fourier map
*R*[*F*^2^ > 2σ(*F*^2^)] = 0.034	Hydrogen site location: inferred from neighbouring sites
*wR*(*F*^2^) = 0.079	H-atom parameters constrained
*S* = 1.06	*w* = 1/[σ^2^(*F*_o_^2^) + (0.0473*P*)^2^] where *P* = (*F*_o_^2^ + 2*F*_c_^2^)/3
8557 reflections	(Δ/σ)_max_ = 0.001
577 parameters	Δρ_max_ = 2.27 e Å^−^^3^
0 restraints	Δρ_min_ = −2.55 e Å^−^^3^

### Special details

**Table:**

Geometry. All esds (except the esd in the dihedral angle between two l.s. planes) are estimated using the full covariance matrix. The cell esds are taken into account individually in the estimation of esds in distances, angles and torsion angles; correlations between esds in cell parameters are only used when they are defined by crystal symmetry. An approximate (isotropic) treatment of cell esds is used for estimating esds involving l.s. planes.
Refinement. Refinement of *F*^2^ against ALL reflections. The weighted *R*-factor wR and goodness of fit *S* are based on *F*^2^, conventional *R*-factors *R* are based on *F*, with *F* set to zero for negative *F*^2^. The threshold expression of *F*^2^ > σ(*F*^2^) is used only for calculating *R*-factors(gt) etc. and is not relevant to the choice of reflections for refinement. *R*-factors based on *F*^2^ are statistically about twice as large as those based on *F*, and *R*- factors based on ALL data will be even larger.

### Fractional atomic coordinates and isotropic or equivalent isotropic displacement parameters (Å^2^)

**Table:**

	*x*	*y*	*z*	*U*_iso_*/*U*_eq_	
Te1A	0.29742 (5)	0.72479 (4)	0.50093 (3)	0.01642 (12)	
Te2A	0.36142 (5)	0.60877 (4)	0.30316 (3)	0.01644 (12)	
Te1B	0.35375 (5)	0.18505 (4)	0.27409 (3)	0.01525 (12)	
Te2B	0.20684 (5)	0.33550 (4)	0.18717 (3)	0.01658 (12)	
Re1A	0.09628 (3)	0.60064 (2)	0.347709 (19)	0.01676 (9)	
Re2A	0.53570 (3)	0.77358 (2)	0.431685 (18)	0.01559 (9)	
Re1B	0.06866 (3)	0.15893 (2)	0.181187 (18)	0.01653 (9)	
Re2B	0.49743 (3)	0.33530 (2)	0.233289 (18)	0.01520 (9)	
O1A	0.1349 (9)	0.4515 (6)	0.4255 (5)	0.058 (2)	
O2A	−0.0895 (7)	0.4533 (5)	0.1750 (4)	0.0373 (17)	
O3A	−0.1911 (7)	0.5972 (5)	0.3997 (4)	0.0400 (18)	
O4A	0.0784 (7)	0.7629 (5)	0.2859 (4)	0.0307 (15)	
O5A	0.6778 (7)	0.6705 (5)	0.5350 (4)	0.0287 (14)	
O6A	0.7801 (7)	0.7870 (5)	0.3370 (4)	0.0345 (16)	
O7A	0.7103 (7)	0.9560 (5)	0.5780 (4)	0.0401 (17)	
O8A	0.3865 (8)	0.8824 (5)	0.3393 (5)	0.0428 (18)	
O1B	0.1637 (7)	0.0630 (5)	0.0225 (4)	0.0319 (16)	
O2B	−0.0790 (8)	−0.0329 (5)	0.1846 (4)	0.0408 (17)	
O3B	−0.2190 (7)	0.1679 (6)	0.0799 (4)	0.046 (2)	
O4B	0.0069 (7)	0.2418 (5)	0.3568 (4)	0.0308 (15)	
O5B	0.4357 (7)	0.2119 (5)	0.0444 (4)	0.0329 (16)	
O6B	0.8091 (7)	0.3224 (6)	0.2827 (5)	0.051 (2)	
O7B	0.6296 (7)	0.5079 (5)	0.1913 (4)	0.0344 (16)	
O8B	0.5370 (8)	0.4615 (5)	0.4184 (4)	0.048 (2)	
C1A	0.1194 (10)	0.5057 (7)	0.3980 (6)	0.033 (2)	
C2A	−0.0188 (9)	0.5080 (7)	0.2378 (6)	0.025 (2)	
C3A	−0.0839 (9)	0.6001 (6)	0.3829 (5)	0.024 (2)	
C4A	0.0871 (8)	0.7024 (6)	0.3063 (5)	0.0190 (19)	
C5A	0.6271 (8)	0.7069 (6)	0.4968 (5)	0.0198 (18)	
C6A	0.6887 (9)	0.7836 (7)	0.3733 (5)	0.023 (2)	
C7A	0.6431 (9)	0.8870 (7)	0.5239 (5)	0.028 (2)	
C8A	0.4374 (9)	0.8407 (7)	0.3710 (5)	0.026 (2)	
C9A	0.2264 (8)	0.8398 (6)	0.5416 (5)	0.0168 (17)	
C10A	0.0903 (9)	0.8416 (7)	0.5024 (5)	0.025 (2)	
H10A	0.0299	0.7943	0.4513	0.030*	
C11A	0.0440 (10)	0.9135 (6)	0.5392 (5)	0.026 (2)	
H11A	−0.0493	0.9138	0.5128	0.031*	
C12A	0.1285 (10)	0.9834 (7)	0.6117 (6)	0.030 (2)	
H12A	0.0944	1.0312	0.6364	0.036*	
C13A	0.2672 (10)	0.9826 (7)	0.6488 (6)	0.032 (2)	
H13A	0.3292	1.0317	0.6982	0.038*	
C14A	0.3154 (10)	0.9112 (7)	0.6144 (5)	0.029 (2)	
H14A	0.4092	0.9114	0.6406	0.035*	
C15A	0.3338 (9)	0.6387 (6)	0.1869 (5)	0.0183 (17)	
C16A	0.4588 (9)	0.6670 (7)	0.1605 (5)	0.027 (2)	
H16A	0.5529	0.6779	0.1956	0.032*	
C17A	0.4462 (10)	0.6795 (7)	0.0813 (5)	0.027 (2)	
H17A	0.5316	0.6999	0.0635	0.033*	
C18A	0.3091 (10)	0.6620 (7)	0.0303 (5)	0.029 (2)	
H18A	0.3004	0.6699	−0.0230	0.035*	
C19A	0.1826 (10)	0.6327 (7)	0.0560 (5)	0.028 (2)	
H19A	0.0883	0.6196	0.0200	0.034*	
C20A	0.1964 (9)	0.6228 (6)	0.1357 (5)	0.024 (2)	
H20A	0.1112	0.6051	0.1545	0.029*	
C1B	0.1288 (9)	0.1010 (6)	0.0792 (5)	0.0227 (19)	
C2B	−0.0234 (9)	0.0381 (6)	0.1836 (5)	0.0220 (19)	
C3B	−0.1129 (9)	0.1624 (7)	0.1170 (5)	0.026 (2)	
C4B	0.0294 (8)	0.2123 (6)	0.2923 (5)	0.0189 (18)	
C5B	0.4600 (9)	0.2567 (6)	0.1133 (5)	0.0206 (19)	
C6B	0.6923 (10)	0.3255 (7)	0.2651 (5)	0.027 (2)	
C7B	0.5788 (9)	0.4447 (7)	0.2070 (5)	0.023 (2)	
C8B	0.5214 (9)	0.4138 (7)	0.3521 (5)	0.027 (2)	
C9B	0.3979 (8)	0.0687 (6)	0.2002 (5)	0.0172 (17)	
C10B	0.5088 (10)	0.0756 (7)	0.1601 (5)	0.028 (2)	
H10B	0.5667	0.1334	0.1621	0.034*	
C11B	0.5364 (11)	−0.0016 (8)	0.1168 (6)	0.037 (2)	
H11B	0.6135	0.0035	0.0897	0.044*	
C12B	0.4503 (12)	−0.0871 (7)	0.1132 (6)	0.036 (2)	
H12B	0.4691	−0.1401	0.0840	0.044*	
C13B	0.3373 (11)	−0.0936 (7)	0.1526 (6)	0.032 (2)	
H13B	0.2755	−0.1515	0.1481	0.039*	
C14B	0.3140 (10)	−0.0152 (6)	0.1991 (5)	0.026 (2)	
H14B	0.2415	−0.0193	0.2295	0.031*	
C15B	0.1889 (8)	0.3331 (6)	0.0598 (5)	0.0185 (18)	
C16B	0.2680 (10)	0.4114 (7)	0.0489 (5)	0.028 (2)	
H16B	0.3248	0.4638	0.0969	0.033*	
C17B	0.2642 (10)	0.4132 (7)	−0.0335 (5)	0.028 (2)	
H17B	0.3195	0.4668	−0.0405	0.034*	
C18B	0.1808 (9)	0.3378 (7)	−0.1040 (5)	0.027 (2)	
H18B	0.1789	0.3394	−0.1593	0.033*	
C19B	0.0998 (10)	0.2595 (7)	−0.0936 (6)	0.031 (2)	
H19B	0.0430	0.2073	−0.1419	0.037*	
C20B	0.1017 (9)	0.2572 (7)	−0.0113 (5)	0.024 (2)	
H20B	0.0438	0.2043	−0.0044	0.029*	

### Atomic displacement parameters (Å^2^)

**Table:**

	*U*^11^	*U*^22^	*U*^33^	*U*^12^	*U*^13^	*U*^23^
Te1A	0.0149 (2)	0.0184 (3)	0.0158 (2)	0.0060 (2)	0.00256 (19)	0.0063 (2)
Te2A	0.0150 (3)	0.0155 (3)	0.0173 (2)	0.0057 (2)	0.00262 (19)	0.0038 (2)
Te1B	0.0183 (3)	0.0135 (3)	0.0139 (2)	0.0054 (2)	0.00404 (19)	0.0049 (2)
Te2B	0.0156 (3)	0.0146 (3)	0.0191 (2)	0.0052 (2)	0.0038 (2)	0.0056 (2)
Re1A	0.01346 (16)	0.0165 (2)	0.01774 (15)	0.00313 (14)	0.00234 (12)	0.00503 (13)
Re2A	0.01336 (16)	0.01502 (19)	0.01671 (15)	0.00443 (14)	0.00232 (12)	0.00443 (13)
Re1B	0.01496 (16)	0.0154 (2)	0.01675 (15)	0.00200 (14)	0.00352 (12)	0.00532 (13)
Re2B	0.01394 (16)	0.0144 (2)	0.01666 (15)	0.00354 (14)	0.00324 (12)	0.00625 (13)
O1A	0.062 (5)	0.044 (6)	0.068 (5)	0.011 (5)	−0.003 (4)	0.037 (5)
O2A	0.029 (4)	0.039 (5)	0.024 (3)	0.003 (3)	−0.003 (3)	−0.004 (3)
O3A	0.025 (4)	0.048 (5)	0.041 (4)	0.004 (3)	0.018 (3)	0.009 (3)
O4A	0.035 (4)	0.025 (4)	0.030 (3)	0.012 (3)	0.002 (3)	0.010 (3)
O5A	0.031 (3)	0.031 (4)	0.029 (3)	0.019 (3)	0.003 (3)	0.014 (3)
O6A	0.027 (3)	0.043 (5)	0.031 (3)	0.010 (3)	0.013 (3)	0.007 (3)
O7A	0.033 (4)	0.028 (5)	0.037 (4)	−0.003 (3)	0.007 (3)	−0.007 (3)
O8A	0.038 (4)	0.040 (5)	0.061 (4)	0.019 (4)	0.005 (3)	0.032 (4)
O1B	0.031 (3)	0.033 (4)	0.021 (3)	0.004 (3)	0.009 (3)	0.000 (3)
O2B	0.053 (4)	0.020 (4)	0.043 (4)	−0.001 (4)	0.015 (3)	0.014 (3)
O3B	0.029 (4)	0.067 (6)	0.044 (4)	0.010 (4)	0.008 (3)	0.030 (4)
O4B	0.037 (4)	0.035 (4)	0.027 (3)	0.018 (3)	0.016 (3)	0.011 (3)
O5B	0.040 (4)	0.035 (4)	0.022 (3)	0.011 (3)	0.014 (3)	0.005 (3)
O6B	0.024 (4)	0.067 (6)	0.075 (5)	0.024 (4)	0.010 (4)	0.039 (5)
O7B	0.029 (4)	0.033 (5)	0.051 (4)	0.009 (3)	0.015 (3)	0.028 (4)
O8B	0.063 (5)	0.031 (5)	0.025 (4)	−0.008 (4)	0.014 (3)	−0.003 (3)
C1A	0.030 (5)	0.036 (7)	0.030 (5)	0.010 (5)	0.003 (4)	0.012 (5)
C2A	0.020 (4)	0.026 (6)	0.035 (5)	0.013 (4)	0.012 (4)	0.014 (4)
C3A	0.023 (5)	0.026 (6)	0.020 (4)	0.005 (4)	0.010 (3)	0.005 (4)
C4A	0.009 (4)	0.027 (6)	0.015 (4)	0.003 (4)	0.001 (3)	0.002 (4)
C5A	0.016 (4)	0.019 (5)	0.020 (4)	0.005 (4)	0.007 (3)	−0.001 (4)
C6A	0.016 (4)	0.031 (6)	0.016 (4)	0.003 (4)	−0.001 (3)	0.005 (4)
C7A	0.019 (4)	0.045 (7)	0.029 (5)	0.014 (5)	0.013 (4)	0.018 (5)
C8A	0.024 (5)	0.023 (6)	0.031 (5)	0.004 (4)	0.011 (4)	0.010 (4)
C9A	0.014 (4)	0.017 (5)	0.023 (4)	0.006 (4)	0.008 (3)	0.011 (4)
C10A	0.028 (5)	0.029 (6)	0.021 (4)	0.013 (4)	0.006 (4)	0.010 (4)
C11A	0.025 (5)	0.019 (5)	0.035 (5)	0.007 (4)	0.007 (4)	0.012 (4)
C12A	0.041 (5)	0.024 (6)	0.033 (5)	0.018 (5)	0.017 (4)	0.010 (4)
C13A	0.031 (5)	0.024 (6)	0.030 (5)	0.006 (5)	0.002 (4)	−0.001 (4)
C14A	0.023 (5)	0.029 (6)	0.028 (4)	0.008 (4)	0.002 (4)	0.004 (4)
C15A	0.024 (4)	0.009 (5)	0.018 (4)	0.007 (4)	0.005 (3)	−0.001 (3)
C16A	0.020 (4)	0.033 (6)	0.030 (4)	0.007 (4)	0.003 (4)	0.018 (4)
C17A	0.032 (5)	0.027 (6)	0.026 (4)	0.008 (5)	0.012 (4)	0.013 (4)
C18A	0.043 (6)	0.032 (6)	0.017 (4)	0.017 (5)	0.009 (4)	0.010 (4)
C19A	0.027 (5)	0.028 (6)	0.024 (4)	0.008 (4)	0.000 (4)	0.007 (4)
C20A	0.027 (5)	0.028 (6)	0.018 (4)	0.014 (4)	0.007 (3)	0.004 (4)
C1B	0.021 (4)	0.022 (5)	0.016 (4)	0.002 (4)	−0.002 (3)	0.005 (4)
C2B	0.020 (4)	0.014 (5)	0.028 (4)	0.002 (4)	0.011 (4)	0.003 (4)
C3B	0.018 (4)	0.032 (6)	0.030 (4)	0.004 (4)	0.001 (4)	0.019 (4)
C4B	0.012 (4)	0.016 (5)	0.027 (4)	0.001 (4)	0.001 (3)	0.013 (4)
C5B	0.018 (4)	0.024 (5)	0.028 (5)	0.011 (4)	0.011 (3)	0.013 (4)
C6B	0.024 (5)	0.029 (6)	0.034 (5)	0.010 (4)	0.009 (4)	0.018 (4)
C7B	0.020 (4)	0.029 (6)	0.028 (4)	0.014 (4)	0.005 (4)	0.014 (4)
C8B	0.026 (5)	0.026 (6)	0.024 (5)	0.002 (4)	0.004 (4)	0.008 (4)
C9B	0.020 (4)	0.018 (5)	0.017 (4)	0.009 (4)	0.004 (3)	0.009 (3)
C10B	0.039 (5)	0.025 (6)	0.032 (5)	0.019 (5)	0.015 (4)	0.015 (4)
C11B	0.049 (6)	0.041 (7)	0.034 (5)	0.025 (6)	0.025 (5)	0.014 (5)
C12B	0.056 (7)	0.028 (6)	0.037 (5)	0.027 (6)	0.023 (5)	0.011 (5)
C13B	0.038 (5)	0.018 (6)	0.039 (5)	0.008 (5)	0.012 (4)	0.008 (4)
C14B	0.031 (5)	0.021 (6)	0.031 (5)	0.012 (4)	0.016 (4)	0.011 (4)
C15B	0.017 (4)	0.021 (5)	0.019 (4)	0.009 (4)	0.003 (3)	0.006 (3)
C16B	0.031 (5)	0.024 (6)	0.027 (4)	0.007 (4)	0.004 (4)	0.012 (4)
C17B	0.029 (5)	0.026 (6)	0.034 (5)	0.006 (5)	0.011 (4)	0.018 (4)
C18B	0.028 (5)	0.038 (6)	0.023 (4)	0.014 (5)	0.007 (4)	0.016 (4)
C19B	0.032 (5)	0.030 (6)	0.031 (5)	0.014 (5)	0.004 (4)	0.010 (4)
C20B	0.017 (4)	0.025 (6)	0.025 (4)	0.002 (4)	−0.001 (3)	0.011 (4)

### Geometric parameters (Å, °)

**Table:**

Te1A—C9A	2.150 (8)	C9A—C14A	1.386 (12)
Te1A—Re1A	2.8115 (7)	C9A—C10A	1.398 (11)
Te1A—Re2A	2.8269 (7)	C10A—C11A	1.395 (12)
Te2A—C15A	2.169 (8)	C10A—H10A	0.9500
Te2A—Re2A	2.8031 (8)	C11A—C12A	1.363 (13)
Te2A—Re1A	2.8131 (7)	C11A—H11A	0.9500
Te1B—C9B	2.158 (8)	C12A—C13A	1.403 (13)
Te1B—Re2B	2.8215 (7)	C12A—H12A	0.9500
Te1B—Re1B	2.8217 (7)	C13A—C14A	1.390 (13)
Te2B—C15B	2.143 (7)	C13A—H13A	0.9500
Te2B—Re1B	2.8030 (7)	C14A—H14A	0.9500
Te2B—Re2B	2.8237 (7)	C15A—C20A	1.387 (11)
Re1A—C2A	1.955 (9)	C15A—C16A	1.388 (12)
Re1A—C3A	1.962 (8)	C16A—C17A	1.416 (11)
Re1A—C4A	2.001 (9)	C16A—H16A	0.9500
Re1A—C1A	2.020 (10)	C17A—C18A	1.377 (13)
Re2A—C7A	1.935 (10)	C17A—H17A	0.9500
Re2A—C6A	1.937 (8)	C18A—C19A	1.396 (13)
Re2A—C8A	2.019 (9)	C18A—H18A	0.9500
Re2A—C5A	2.026 (9)	C19A—C20A	1.405 (11)
Re1B—C3B	1.947 (8)	C19A—H19A	0.9500
Re1B—C2B	1.954 (9)	C20A—H20A	0.9500
Re1B—C1B	2.000 (8)	C9B—C10B	1.378 (11)
Re1B—C4B	2.001 (8)	C9B—C14B	1.390 (12)
Re2B—C6B	1.957 (9)	C10B—C11B	1.390 (13)
Re2B—C7B	1.963 (9)	C10B—H10B	0.9500
Re2B—C5B	1.994 (8)	C11B—C12B	1.402 (15)
Re2B—C8B	2.004 (9)	C11B—H11B	0.9500
O1A—C1A	1.148 (12)	C12B—C13B	1.390 (13)
O2A—C2A	1.138 (11)	C12B—H12B	0.9500
O3A—C3A	1.129 (10)	C13B—C14B	1.401 (13)
O4A—C4A	1.157 (11)	C13B—H13B	0.9500
O5A—C5A	1.138 (10)	C14B—H14B	0.9500
O6A—C6A	1.174 (10)	C15B—C16B	1.389 (12)
O7A—C7A	1.164 (12)	C15B—C20B	1.400 (12)
O8A—C8A	1.146 (11)	C16B—C17B	1.411 (11)
O1B—C1B	1.152 (10)	C16B—H16B	0.9500
O2B—C2B	1.147 (11)	C17B—C18B	1.383 (13)
O3B—C3B	1.162 (10)	C17B—H17B	0.9500
O4B—C4B	1.155 (9)	C18B—C19B	1.390 (13)
O5B—C5B	1.139 (10)	C18B—H18B	0.9500
O6B—C6B	1.151 (10)	C19B—C20B	1.415 (12)
O7B—C7B	1.147 (11)	C19B—H19B	0.9500
O8B—C8B	1.134 (11)	C20B—H20B	0.9500
			
C9A—Te1A—Re1A	108.5 (2)	C14A—C9A—C10A	119.4 (8)
C9A—Te1A—Re2A	108.5 (2)	C14A—C9A—Te1A	117.5 (6)
Re1A—Te1A—Re2A	96.25 (2)	C10A—C9A—Te1A	122.9 (6)
C15A—Te2A—Re2A	105.6 (2)	C11A—C10A—C9A	119.4 (8)
C15A—Te2A—Re1A	105.0 (2)	C11A—C10A—H10A	120.3
Re2A—Te2A—Re1A	96.76 (2)	C9A—C10A—H10A	120.3
C9B—Te1B—Re2B	107.9 (2)	C12A—C11A—C10A	122.1 (8)
C9B—Te1B—Re1B	102.1 (2)	C12A—C11A—H11A	119.0
Re2B—Te1B—Re1B	95.74 (2)	C10A—C11A—H11A	119.0
C15B—Te2B—Re1B	108.3 (2)	C11A—C12A—C13A	118.1 (8)
C15B—Te2B—Re2B	100.4 (2)	C11A—C12A—H12A	120.9
Re1B—Te2B—Re2B	96.11 (2)	C13A—C12A—H12A	120.9
C2A—Re1A—C3A	90.7 (3)	C14A—C13A—C12A	121.1 (9)
C2A—Re1A—C4A	92.9 (3)	C14A—C13A—H13A	119.4
C3A—Re1A—C4A	89.8 (3)	C12A—C13A—H13A	119.4
C2A—Re1A—C1A	91.8 (4)	C9A—C14A—C13A	119.8 (8)
C3A—Re1A—C1A	91.4 (4)	C9A—C14A—H14A	120.1
C4A—Re1A—C1A	175.2 (4)	C13A—C14A—H14A	120.1
C2A—Re1A—Te1A	171.3 (2)	C20A—C15A—C16A	120.0 (7)
C3A—Re1A—Te1A	97.4 (2)	C20A—C15A—Te2A	122.3 (6)
C4A—Re1A—Te1A	90.4 (2)	C16A—C15A—Te2A	117.4 (6)
C1A—Re1A—Te1A	84.8 (3)	C15A—C16A—C17A	120.0 (8)
C2A—Re1A—Te2A	91.8 (2)	C15A—C16A—H16A	120.0
C3A—Re1A—Te2A	177.5 (3)	C17A—C16A—H16A	120.0
C4A—Re1A—Te2A	89.3 (2)	C18A—C17A—C16A	119.5 (8)
C1A—Re1A—Te2A	89.2 (3)	C18A—C17A—H17A	120.2
Te1A—Re1A—Te2A	80.23 (2)	C16A—C17A—H17A	120.2
C7A—Re2A—C6A	93.9 (4)	C17A—C18A—C19A	120.8 (8)
C7A—Re2A—C8A	89.2 (4)	C17A—C18A—H18A	119.6
C6A—Re2A—C8A	92.4 (3)	C19A—C18A—H18A	119.6
C7A—Re2A—C5A	90.1 (4)	C18A—C19A—C20A	119.3 (8)
C6A—Re2A—C5A	90.6 (3)	C18A—C19A—H19A	120.4
C8A—Re2A—C5A	176.9 (3)	C20A—C19A—H19A	120.4
C7A—Re2A—Te2A	175.6 (2)	C15A—C20A—C19A	120.3 (8)
C6A—Re2A—Te2A	90.5 (3)	C15A—C20A—H20A	119.8
C8A—Re2A—Te2A	90.0 (3)	C19A—C20A—H20A	119.8
C5A—Re2A—Te2A	90.5 (2)	O1B—C1B—Re1B	175.4 (8)
C7A—Re2A—Te1A	95.6 (2)	O2B—C2B—Re1B	179.2 (8)
C6A—Re2A—Te1A	169.6 (3)	O3B—C3B—Re1B	177.5 (9)
C8A—Re2A—Te1A	92.0 (2)	O4B—C4B—Re1B	178.7 (7)
C5A—Re2A—Te1A	85.1 (2)	O5B—C5B—Re2B	178.6 (7)
Te2A—Re2A—Te1A	80.14 (2)	O6B—C6B—Re2B	177.4 (8)
C3B—Re1B—C2B	93.9 (4)	O7B—C7B—Re2B	178.1 (7)
C3B—Re1B—C1B	95.1 (3)	O8B—C8B—Re2B	177.0 (9)
C2B—Re1B—C1B	88.5 (3)	C10B—C9B—C14B	120.5 (8)
C3B—Re1B—C4B	92.5 (3)	C10B—C9B—Te1B	123.1 (7)
C2B—Re1B—C4B	89.0 (3)	C14B—C9B—Te1B	116.3 (5)
C1B—Re1B—C4B	172.1 (3)	C9B—C10B—C11B	120.3 (9)
C3B—Re1B—Te2B	88.0 (3)	C9B—C10B—H10B	119.8
C2B—Re1B—Te2B	176.9 (2)	C11B—C10B—H10B	119.8
C1B—Re1B—Te2B	93.7 (3)	C10B—C11B—C12B	120.0 (9)
C4B—Re1B—Te2B	88.5 (2)	C10B—C11B—H11B	120.0
C3B—Re1B—Te1B	169.9 (3)	C12B—C11B—H11B	120.0
C2B—Re1B—Te1B	96.2 (2)	C13B—C12B—C11B	119.3 (9)
C1B—Re1B—Te1B	85.5 (2)	C13B—C12B—H12B	120.3
C4B—Re1B—Te1B	87.3 (2)	C11B—C12B—H12B	120.3
Te2B—Re1B—Te1B	81.86 (2)	C12B—C13B—C14B	120.4 (9)
C6B—Re2B—C7B	91.7 (4)	C12B—C13B—H13B	119.8
C6B—Re2B—C5B	92.2 (3)	C14B—C13B—H13B	119.8
C7B—Re2B—C5B	91.1 (4)	C9B—C14B—C13B	119.4 (8)
C6B—Re2B—C8B	91.4 (4)	C9B—C14B—H14B	120.3
C7B—Re2B—C8B	89.3 (4)	C13B—C14B—H14B	120.3
C5B—Re2B—C8B	176.3 (3)	C16B—C15B—C20B	119.5 (7)
C6B—Re2B—Te1B	94.2 (3)	C16B—C15B—Te2B	117.4 (6)
C7B—Re2B—Te1B	173.5 (2)	C20B—C15B—Te2B	123.1 (6)
C5B—Re2B—Te1B	91.3 (2)	C15B—C16B—C17B	120.1 (9)
C8B—Re2B—Te1B	87.9 (3)	C15B—C16B—H16B	119.9
C6B—Re2B—Te2B	175.5 (3)	C17B—C16B—H16B	119.9
C7B—Re2B—Te2B	92.6 (2)	C18B—C17B—C16B	120.7 (9)
C5B—Re2B—Te2B	86.6 (2)	C18B—C17B—H17B	119.7
C8B—Re2B—Te2B	89.7 (3)	C16B—C17B—H17B	119.7
Te1B—Re2B—Te2B	81.502 (19)	C17B—C18B—C19B	119.6 (8)
O1A—C1A—Re1A	178.4 (9)	C17B—C18B—H18B	120.2
O2A—C2A—Re1A	177.7 (8)	C19B—C18B—H18B	120.2
O3A—C3A—Re1A	176.9 (8)	C18B—C19B—C20B	120.2 (9)
O4A—C4A—Re1A	176.2 (7)	C18B—C19B—H19B	119.9
O5A—C5A—Re2A	178.4 (7)	C20B—C19B—H19B	119.9
O6A—C6A—Re2A	178.0 (9)	C15B—C20B—C19B	119.9 (9)
O7A—C7A—Re2A	178.1 (8)	C15B—C20B—H20B	120.0
O8A—C8A—Re2A	176.8 (9)	C19B—C20B—H20B	120.0
			
C9A—Te1A—Re1A—C2A	154.5 (18)	Te1A—Re2A—C5A—O5A	−51 (28)
Re2A—Te1A—Re1A—C2A	42.6 (17)	C7A—Re2A—C6A—O6A	152 (20)
C9A—Te1A—Re1A—C3A	−48.0 (3)	C8A—Re2A—C6A—O6A	−119 (20)
Re2A—Te1A—Re1A—C3A	−159.9 (3)	C5A—Re2A—C6A—O6A	61 (20)
C9A—Te1A—Re1A—C4A	41.9 (3)	Te2A—Re2A—C6A—O6A	−29 (20)
Re2A—Te1A—Re1A—C4A	−70.0 (2)	Te1A—Re2A—C6A—O6A	−4(21)
C9A—Te1A—Re1A—C1A	−138.7 (3)	C6A—Re2A—C7A—O7A	18 (25)
Re2A—Te1A—Re1A—C1A	109.4 (3)	C8A—Re2A—C7A—O7A	−74 (25)
C9A—Te1A—Re1A—Te2A	131.2 (2)	C5A—Re2A—C7A—O7A	109 (25)
Re2A—Te1A—Re1A—Te2A	19.292 (18)	Te2A—Re2A—C7A—O7A	−154 (22)
C15A—Te2A—Re1A—C2A	55.8 (3)	Te1A—Re2A—C7A—O7A	−166 (25)
Re2A—Te2A—Re1A—C2A	164.0 (3)	C7A—Re2A—C8A—O8A	13 (14)
C15A—Te2A—Re1A—C3A	−108 (6)	C6A—Re2A—C8A—O8A	−81 (14)
Re2A—Te2A—Re1A—C3A	0(6)	C5A—Re2A—C8A—O8A	88 (15)
C15A—Te2A—Re1A—C4A	−37.1 (3)	Te2A—Re2A—C8A—O8A	−171 (14)
Re2A—Te2A—Re1A—C4A	71.1 (2)	Te1A—Re2A—C8A—O8A	108 (14)
C15A—Te2A—Re1A—C1A	147.5 (4)	Re1A—Te1A—C9A—C14A	−176.9 (6)
Re2A—Te2A—Re1A—C1A	−104.3 (3)	Re2A—Te1A—C9A—C14A	−73.5 (6)
C15A—Te2A—Re1A—Te1A	−127.6 (2)	Re1A—Te1A—C9A—C10A	8.9 (7)
Re2A—Te2A—Re1A—Te1A	−19.483 (18)	Re2A—Te1A—C9A—C10A	112.3 (6)
C15A—Te2A—Re2A—C7A	114 (4)	C14A—C9A—C10A—C11A	−2.5 (13)
Re1A—Te2A—Re2A—C7A	6(4)	Te1A—C9A—C10A—C11A	171.6 (6)
C15A—Te2A—Re2A—C6A	−57.4 (3)	C9A—C10A—C11A—C12A	1.1 (14)
Re1A—Te2A—Re2A—C6A	−165.1 (3)	C10A—C11A—C12A—C13A	1.2 (14)
C15A—Te2A—Re2A—C8A	35.0 (3)	C11A—C12A—C13A—C14A	−2.2 (14)
Re1A—Te2A—Re2A—C8A	−72.6 (2)	C10A—C9A—C14A—C13A	1.6 (13)
C15A—Te2A—Re2A—C5A	−148.0 (3)	Te1A—C9A—C14A—C13A	−172.8 (7)
Re1A—Te2A—Re2A—C5A	104.3 (2)	C12A—C13A—C14A—C9A	0.8 (14)
C15A—Te2A—Re2A—Te1A	127.1 (2)	Re2A—Te2A—C15A—C20A	−121.9 (7)
Re1A—Te2A—Re2A—Te1A	19.378 (17)	Re1A—Te2A—C15A—C20A	−20.3 (7)
C9A—Te1A—Re2A—C7A	47.8 (3)	Re2A—Te2A—C15A—C16A	64.0 (7)
Re1A—Te1A—Re2A—C7A	159.6 (3)	Re1A—Te2A—C15A—C16A	165.7 (6)
C9A—Te1A—Re2A—C6A	−156.6 (13)	C20A—C15A—C16A—C17A	0.0 (14)
Re1A—Te1A—Re2A—C6A	−44.8 (13)	Te2A—C15A—C16A—C17A	174.2 (7)
C9A—Te1A—Re2A—C8A	−41.6 (3)	C15A—C16A—C17A—C18A	−1.1 (14)
Re1A—Te1A—Re2A—C8A	70.3 (3)	C16A—C17A—C18A—C19A	0.5 (15)
C9A—Te1A—Re2A—C5A	137.4 (3)	C17A—C18A—C19A—C20A	1.2 (15)
Re1A—Te1A—Re2A—C5A	−110.8 (2)	C16A—C15A—C20A—C19A	1.8 (13)
C9A—Te1A—Re2A—Te2A	−131.2 (2)	Te2A—C15A—C20A—C19A	−172.2 (7)
Re1A—Te1A—Re2A—Te2A	−19.370 (18)	C18A—C19A—C20A—C15A	−2.4 (14)
C15B—Te2B—Re1B—C3B	−60.3 (3)	C3B—Re1B—C1B—O1B	−123 (9)
Re2B—Te2B—Re1B—C3B	−163.4 (3)	C2B—Re1B—C1B—O1B	−29 (9)
C15B—Te2B—Re1B—C2B	171 (4)	C4B—Re1B—C1B—O1B	43 (10)
Re2B—Te2B—Re1B—C2B	68 (4)	Te2B—Re1B—C1B—O1B	149 (9)
C15B—Te2B—Re1B—C1B	34.7 (3)	Te1B—Re1B—C1B—O1B	67 (9)
Re2B—Te2B—Re1B—C1B	−68.4 (2)	C3B—Re1B—C2B—O2B	−3(60)
C15B—Te2B—Re1B—C4B	−152.9 (3)	C1B—Re1B—C2B—O2B	−98 (60)
Re2B—Te2B—Re1B—C4B	104.0 (2)	C4B—Re1B—C2B—O2B	89 (60)
C15B—Te2B—Re1B—Te1B	119.6 (2)	Te2B—Re1B—C2B—O2B	125 (57)
Re2B—Te2B—Re1B—Te1B	16.538 (16)	Te1B—Re1B—C2B—O2B	176 (100)
C9B—Te1B—Re1B—C3B	−125.8 (15)	C2B—Re1B—C3B—O3B	158 (17)
Re2B—Te1B—Re1B—C3B	−16.1 (15)	C1B—Re1B—C3B—O3B	−113 (17)
C9B—Te1B—Re1B—C2B	56.1 (3)	C4B—Re1B—C3B—O3B	69 (17)
Re2B—Te1B—Re1B—C2B	165.9 (2)	Te2B—Re1B—C3B—O3B	−20 (17)
C9B—Te1B—Re1B—C1B	−31.9 (3)	Te1B—Re1B—C3B—O3B	−20 (18)
Re2B—Te1B—Re1B—C1B	77.9 (3)	C3B—Re1B—C4B—O4B	123 (32)
C9B—Te1B—Re1B—C4B	144.8 (3)	C2B—Re1B—C4B—O4B	29 (32)
Re2B—Te1B—Re1B—C4B	−105.4 (2)	C1B—Re1B—C4B—O4B	−42 (33)
C9B—Te1B—Re1B—Te2B	−126.3 (2)	Te2B—Re1B—C4B—O4B	−149 (32)
Re2B—Te1B—Re1B—Te2B	−16.540 (17)	Te1B—Re1B—C4B—O4B	−67 (32)
C9B—Te1B—Re2B—C6B	−57.6 (3)	C6B—Re2B—C5B—O5B	173 (100)
Re1B—Te1B—Re2B—C6B	−162.3 (3)	C7B—Re2B—C5B—O5B	−95 (32)
C9B—Te1B—Re2B—C7B	147 (2)	C8B—Re2B—C5B—O5B	2(36)
Re1B—Te1B—Re2B—C7B	42 (2)	Te1B—Re2B—C5B—O5B	79 (32)
C9B—Te1B—Re2B—C5B	34.7 (3)	Te2B—Re2B—C5B—O5B	−2(32)
Re1B—Te1B—Re2B—C5B	−70.0 (2)	C7B—Re2B—C6B—O6B	−8(20)
C9B—Te1B—Re2B—C8B	−148.9 (3)	C5B—Re2B—C6B—O6B	84 (20)
Re1B—Te1B—Re2B—C8B	106.4 (3)	C8B—Re2B—C6B—O6B	−97 (20)
C9B—Te1B—Re2B—Te2B	121.1 (2)	Te1B—Re2B—C6B—O6B	175 (100)
Re1B—Te1B—Re2B—Te2B	16.430 (17)	Te2B—Re2B—C6B—O6B	159 (18)
C15B—Te2B—Re2B—C6B	−110 (3)	C6B—Re2B—C7B—O7B	22 (25)
Re1B—Te2B—Re2B—C6B	0(3)	C5B—Re2B—C7B—O7B	−71 (25)
C15B—Te2B—Re2B—C7B	56.3 (4)	C8B—Re2B—C7B—O7B	113 (25)
Re1B—Te2B—Re2B—C7B	166.2 (3)	Te1B—Re2B—C7B—O7B	177 (100)
C15B—Te2B—Re2B—C5B	−34.6 (3)	Te2B—Re2B—C7B—O7B	−157 (25)
Re1B—Te2B—Re2B—C5B	75.3 (2)	C6B—Re2B—C8B—O8B	95 (16)
C15B—Te2B—Re2B—C8B	145.7 (4)	C7B—Re2B—C8B—O8B	4(16)
Re1B—Te2B—Re2B—C8B	−104.4 (3)	C5B—Re2B—C8B—O8B	−93 (16)
C15B—Te2B—Re2B—Te1B	−126.4 (2)	Te1B—Re2B—C8B—O8B	−170 (16)
Re1B—Te2B—Re2B—Te1B	−16.555 (16)	Te2B—Re2B—C8B—O8B	−89 (16)
C2A—Re1A—C1A—O1A	74 (36)	Re2B—Te1B—C9B—C10B	16.1 (7)
C3A—Re1A—C1A—O1A	165 (36)	Re1B—Te1B—C9B—C10B	116.3 (6)
C4A—Re1A—C1A—O1A	−90 (36)	Re2B—Te1B—C9B—C14B	−168.0 (5)
Te1A—Re1A—C1A—O1A	−98 (36)	Re1B—Te1B—C9B—C14B	−67.8 (6)
Te2A—Re1A—C1A—O1A	−17 (36)	C14B—C9B—C10B—C11B	1.1 (13)
C3A—Re1A—C2A—O2A	−23 (21)	Te1B—C9B—C10B—C11B	176.9 (7)
C4A—Re1A—C2A—O2A	−112 (21)	C9B—C10B—C11B—C12B	0.6 (14)
C1A—Re1A—C2A—O2A	69 (21)	C10B—C11B—C12B—C13B	0.4 (14)
Te1A—Re1A—C2A—O2A	135 (20)	C11B—C12B—C13B—C14B	−3.1 (14)
Te2A—Re1A—C2A—O2A	158 (21)	C10B—C9B—C14B—C13B	−3.7 (12)
C2A—Re1A—C3A—O3A	−4(17)	Te1B—C9B—C14B—C13B	−179.7 (6)
C4A—Re1A—C3A—O3A	89 (17)	C12B—C13B—C14B—C9B	4.7 (13)
C1A—Re1A—C3A—O3A	−96 (17)	Re1B—Te2B—C15B—C16B	−171.2 (6)
Te1A—Re1A—C3A—O3A	179 (100)	Re2B—Te2B—C15B—C16B	−71.2 (6)
Te2A—Re1A—C3A—O3A	160 (12)	Re1B—Te2B—C15B—C20B	9.0 (7)
C2A—Re1A—C4A—O4A	123 (11)	Re2B—Te2B—C15B—C20B	109.1 (6)
C3A—Re1A—C4A—O4A	33 (11)	C20B—C15B—C16B—C17B	−2.1 (12)
C1A—Re1A—C4A—O4A	−72 (12)	Te2B—C15B—C16B—C17B	178.1 (6)
Te1A—Re1A—C4A—O4A	−65 (11)	C15B—C16B—C17B—C18B	0.7 (13)
Te2A—Re1A—C4A—O4A	−145 (11)	C16B—C17B—C18B—C19B	0.1 (13)
C7A—Re2A—C5A—O5A	45 (28)	C17B—C18B—C19B—C20B	0.5 (13)
C6A—Re2A—C5A—O5A	139 (28)	C16B—C15B—C20B—C19B	2.8 (12)
C8A—Re2A—C5A—O5A	−31 (32)	Te2B—C15B—C20B—C19B	−177.5 (6)
Te2A—Re2A—C5A—O5A	−131 (28)	C18B—C19B—C20B—C15B	−2.0 (13)

### Hydrogen-bond geometry (Å, °)

**Table:**

*D*—H···*A*	*D*—H	H···*A*	*D*···*A*	*D*—H···*A*
C16A—H16A···O6A	0.95	2.74	3.592 (11)	149
C20A—H20A···O2A	0.95	2.87	3.678 (12)	143
C12B—H12B···O5B^i^	0.95	2.63	3.433 (11)	143

Symmetry codes:  (i) −*x*+1, −*y*, −*z*.
